# Respiratory symptoms and disease characteristics as predictors of pulmonary function abnormalities in patients with rheumatoid arthritis: an observational cohort study

**DOI:** 10.1186/ar3037

**Published:** 2010-05-27

**Authors:** Dimitrios A Pappas, Jon T Giles, Geoffrey Connors, Noah Lechtzin, Joan M Bathon, Sonye K Danoff

**Affiliations:** 1 Division of Pulmonary and Critical Care Medicine, Johns Hopkins University, 1830 E. Monument Street, 5th Floor, Baltimore, MD 21202, USA

## Abstract

**Introduction:**

Lung involvement is a common extra-articular manifestation of rheumatoid arthritis (RA) that confers significant morbidity and mortality. The objective of the present study is to assess which respiratory symptoms and patient and disease characteristics are most highly associated with pulmonary function test (PFT) abnormalities in an RA patient cohort without clinical cardiovascular disease.

**Methods:**

A total of 159 individuals with RA and without clinically evident cardiovascular disease were evaluated. Respiratory symptoms were assessed with the Lung Tissue Research Consortium questionnaire and all patients underwent evaluation with PFTs. Demographic, lifestyle, RA disease and treatment characteristics were collected. Subclinical coronary artery disease was assessed by cardiac computed tomography. Multivariable regression analysis was used to identify pulmonary symptoms and nonpulmonary parameters associated with PFT abnormalities. Areas under the receiver operating characteristic curves (AUC) were calculated to evaluate the discrimination of these variables for identifying patients with PFT abnormalities.

**Results:**

Respiratory symptoms were reported by 42% of the patient population. Although only 6% carried a prior diagnosis of lung disease, PFT abnormalities were identified in 28% of the subjects. Symptoms combined with other patient and RA characteristics (body mass index, current smoking, anti-cyclic citrullinated peptide antibodies, and current prednisone use) performed satisfactorily in predicting the PFT abnormalities of obstruction (AUC = 0.91, 95% confidence interval = 0.78 to 0.98), restriction (AUC = 0.79, 95% confidence interval = 0.75 to 0.93) and impaired diffusion (AUC = 0.85, 95% confidence interval = 0.59 to 0.92). Co-morbid subclinical coronary artery disease did not modify these relationships.

**Conclusions:**

Assessment of respiratory symptoms along with a limited number of clinical parameters may serve as a useful and inexpensive clinical tool for identifying RA patients in need of further pulmonary investigation.

## Introduction

Rheumatoid arthritis (RA) is a highly inflammatory chronic disease associated with diminished physical function and premature mortality [1-5]. Lung involvement is a common extra-articular manifestation of RA [6], conferring significant morbidity and mortality [7]. Interstitial lung disease (ILD) is the most common and most serious form of lung involvement in RA. The reported prevalence of subclinical and clinically evident ILD in RA varies depending on the method of detection, and ranges between 1 and 58% [8-12]. Radiographic changes and changes on pulmonary function testing may precede overt symptoms by years. Once clinically apparent, ILD is associated with significant mortality [13]. Respiratory diseases (including ILD) are a leading cause of excess death in patients with RA [14].

Chronic obstructive pulmonary disease has also been reported to occur more frequently in patients with RA than in the general population after adjusting for smoking, and to have a greater impact on survival [15,16]. Both restrictive lung disease (ILD) and obstructive lung disease thus produce clinically important effects in patients with RA.

Given the impact of lung disease on morbidity and mortality in RA, screening of asymptomatic RA patients for pulmonary involvement has been recommended by some experts [17-19]. The most sensitive method for detecting ILD is high-resolution computed tomography (HRCT) of the chest - but this technique is expensive and associated with significant radiation exposure [20], limiting its suitability for screening of asymptomatic individuals. Pulmonary function testing has proved valuable in early detection of RA-associated lung disease. Nevertheless, the cost of screening all asymptomatic RA patients with pulmonary function tests (PFTs) makes this approach untenable. Clinicians may therefore rely on development of overt respiratory symptoms (for example, dyspnea or cough) and/or physical findings (for example, basilar crackles) in RA patients as the trigger for evaluation for lung disease, an approach endorsed by the British Society of Rheumatology [19,21]. This approach has limited the understanding of the natural history of RA-associated lung disease by identifying patients primarily later in disease, and has contributed to the difficulty in assessing therapeutic agents for lung disease. Further, respiratory symptoms are not specific for pulmonary disease and could represent cardiovascular disease (CVD). Cardiovascular events, including myocardial infarctions and congestive heart failure, are increased twofold to fourfold in RA patients compared with matched non-RA controls [3,5,22,23].

Several studies have evaluated associations between patient-reported respiratory symptoms and lung involvement in RA with conflicting results [10,12,17]. Overall, these studies suggest that pulmonary complaints, physical findings and certain RA-related or other patient characteristics may be more common in patients with documented lung disease than in patients without. These studies largely focused on individual predictors rather than multifactor prediction model, on restrictive disease only, and on structural (HRCT) rather than physiological (PFT) outcome [10,12,17]. Further, these studies did not take into account potential confounding by co-morbid CVD [10,12,17].

We investigated the association of systemically assessed respiratory symptoms and patient and RA-related variables with impaired pulmonary function in a well-characterized cohort of 159 RA patients free of clinically evident CVD. We evaluated whether subclinical coronary artery disease (CAD) may confound these relationships. We sought to identify respiratory symptoms and additional patient characteristics that, alone or in combination, discriminated patients with PFT abnormalities.

## Materials and methods

### Participants and enrollment

The study subjects were participants in the Evaluation of Subclinical Cardiovascular Disease and Predictors of Events in Rheumatoid Arthritis, a cohort study investigating the prevalence, progression, and risk factors for subclinical CVD in RA patients described previously [24]. Participants were 45 to 84 years of age at enrollment and met the American College of Rheumatology (formerly the American Rheumatism Association) 1987 classification criteria for RA [25]. Exclusion criteria were known prior CVD, defined as a prior history of self-reported physician-diagnosed myocardial infarction, heart failure, coronary artery revascularization, angioplasty, peripheral vascular disease or procedures (excluding varicose vein procedures), implanted pacemaker or defibrillator devices, and current atrial fibrillation. The study was approved by the Institutional Review Board of the Johns Hopkins Hospital, with all subjects providing written informed consent.

### Assessments

All clinical and PFT data utilized for this study were from Visit 2, which took place approximately 18 months after the baseline visit. The evaluation for coronary artery calcification took place at the baseline visit.

#### Measurement of the primary outcome

Pulmonary function testing constituted the primary outcome and included spirometry, lung volumes and diffusion capacity according to American Thoracic Society criteria [26]. Obstruction was defined as a forced expiratory volume in the first second/forced vital capacity ratio ≤90% of the predicted ratio. Restriction was defined as a forced vital capacity < 80% of predicted in the absence of concomitant obstructive abnormality. Isolated impaired diffusion capacity was defined by a diffusion capacity < 80% of predicted, in the absence of obstruction or restriction. One hundred and eighty patients completed Visit 2. Of these patients, 159 (88%) had pulmonary function testing completed. The remaining 21 patients were unable to be tested due to logistical considerations.

#### Assessment of respiratory symptoms

The Lung Tissue Research Consortium questionnaire (Additional file 1) [27] was administered by an interviewer during Visit 2 to assess respiratory symptoms. This form includes Items 7 to 13 from the American Thoracic Society Division of Lung Diseases Questionnaire (ATS-DLD-78-C) [28], focusing on cough, phlegm, wheezing and dyspnea. This questionnaire meets American Thoracic Society criteria for epidemiologic surveys in chronic respiratory diseases [29] and is considered reproducible, valid and free of bias [28].

#### Measurement of subclinical cardiovascular disease

Subclinical CAD was assessed at the baseline visit by cardiac multi-row detector computed tomography according to a standard previously published methodology [30]. Coronary artery calcium was quantified using the Agatston method [31]. A phantom of known calcium density was scanned simultaneously with the patient for standardization [32]. Intra-observer and inter-observer agreement for computed tomography assessments has been evaluated and found to be high (kappa = 0.93 and 0.90, respectively) [33]. An Agatston coronary calcium score ≥100, shown to correlate with the presence of plaque and moderate risk for future cardiovascular events, was used as a cut-off value to identify patients with significant subclinical CAD [34,35].

#### Pre-existing diagnoses of lung disease

At the time of PFT and respiratory symptom assessment (Visit 2), patients were asked: 'Has a doctor told you that you have developed any of the following: emphysema, asthma. Has a physician ever diagnosed you with rheumatoid lung disease (other than pulmonary nodules).' Patient-reported diagnoses were not independently verified.

#### Demographic and lifestyle covariates

Demographic and lifestyle characteristics, including current or past smoking, were collected via examiner-administered questionnaires. Physical function was assessed with detailed questions investigating the amount and intensity of intentional exercise and was calculated in minutes/week and metabolic equivalents/week. Functional levels were assessed using the Stanford Health Assessment Questionnaire (HAQ) score [36]. The body mass index (BMI) was calculated as kilograms per meter^2^.

#### RA-specific covariates

RA disease duration was calculated from the date of diagnosis. The RA Disease Activity Score in 28 joints was calculated, including C-reactive protein [37]. Joints were examined for swelling and tenderness by a single trained examiner. The extent of radiographic joint damage was assessed at the baseline visit by a trained radiologist using the modified Sharp Score [38]. Information regarding treatment with biologic and nonbiologic disease-modifying antirheumatic drugs and steroids was collected via detailed questionnaires. All current medication was brought to Visit 2 and the medications and dosages were recorded by research staff.

#### Laboratory covariates

Fasting sera and plasma were stored at -70°C. Serum C-reactive protein levels were measured by high-sensitivity nephelometry (Dade Behring, Deerfield, IL, USA). Rheumatoid factor (RF) and anti-cyclic citrullinated peptide (anti-CCP) antibodies were determined by ELISA with cut-off values for seropositivity being ≥40 units and ≥60 units, respectively.

### Statistical analysis

In initial exploratory data analysis, the distributions of all variables were examined. Means and standard deviations were calculated for normally distributed continuous variables, medians and interquartile ranges for non-normally distributed continuous variables, and counts and percentages calculated for categorical variables. Differences in patient characteristics for the group with abnormal PFTs were compared versus the group with normal PFTs using *t *tests for means, the Kruskal-Wallis test for medians, and the chi-square goodness-of-fit or Fisher's exact test (as appropriate) for proportions.

Receiver operator characteristic (ROC) curves were constructed to examine the ability of pulmonary symptoms to predict PFT abnormalities. Informative tests are defined by an area under the curve (AUC) for a resulting ROC function ≥0.5. Predictors that classify every individual within the sample perfectly result in an AUC of 1.0 [39]. The ROC curve was considered internally valid if the 95% confidence interval (CI) using the bootstrap method did not contain 0.5 (that is, < 50% chance of being uninformative) [40].

Multivariable logistic regression models were expanded to include the pulmonary symptoms from each category (cough, phlegm, wheezing, and breathlessness) with the highest AUC from the univariate models, as well as sociodemographic characteristics, measures of coronary calcification, RA disease and treatment characteristics, and patient report of prior lung disease. Simplified models including only pertinent predictors of PFT outcomes were obtained using hierarchical modeling techniques, with the likelihood ratio test used to exclude predictors lacking any contribution to the variability of the outcome.

From the variables identified in the final predictive models, ROC curves were constructed for models including only reported symptoms, including symptoms + pertinent sociodemographic and RA characteristics, and including symptoms + pertinent characteristics + self-reported lung disease. AUCs for these correlated ROC curves were compared using the nonparametric method of DeLong and colleagues [41]. Two correlated ROC curves with statistically different AUCs indicate that the additional covariates included in the model significantly improved the performance of the model to discriminate the outcome.

Statistical calculations were performed using Intercooled Stata 10.1 (StataCorp, College Station, TX, USA). In all tests, a two-tailed α level of 0.05 was defined as the level of statistical significance.

## Results

The mean age of the cohort was 61 years. The majority of patients were Caucasian (86%) and female (61%), and the median duration of disease was 10.7 years. Two-thirds of the patients were positive for RF (62%) and for CCP (66%). Disease activity in the cohort was mild to moderate (median RA Disease Activity Score in 28 joints score = 3.1) and the mean HAQ score (0.75) indicated mild disability. The majority of patients were receiving disease-modifying antirheumatic drugs - methotrexate being most common (68%), followed by TNF inhibitors (42%) and prednisone (37%). Ninety-two patients (58%) in the cohort had a history of smoking.

At the time of the present analysis, 158 patients in the cohort had completed PFT testing: 45 (28%) demonstrated at least one predefined abnormality. Restrictive lung disease was observed in 12 patients (7.6%) (median forced vital capacity = 71% of predicted; range 63 to 76%), obstructive lung disease in 18 patients (11.3%) (median forced expiratory volume in the first second/forced vital capacity ratio = 85% of predicted ratio; range 79 to 88%) and impaired diffusing capacity in 31 patients (19.8%). Impaired diffusion in conjunction with restriction or obstruction was observed in 16 of those latter subjects, while the remaining 15 patients (9.6%) had isolated impaired diffusion (median diffusion capacity of the lung for carbon monoxide = 69% of predicted, range 59 to 75%). We found no differences in general demographics, pulmonary symptoms or medication use between the 38 patients who did not undergo testing compared with those who did. There was a higher rate of reported prior diagnosis of emphysema and a higher frequency of positive CCP and RF among patients who did not undergo PFT testing.

Patient characteristics according to the presence of any PFT abnormalities are summarized in Table 1. Only 17 (37.8%) of the 45 patients with abnormal PFTs reported a prior known diagnosis of emphysema (eight patients), asthma (eight patients) or rheumatoid lung disease (two patients). Demographic characteristics did not differ among those with and without PFT abnormalities; however, the proportion of current smokers was almost threefold higher in patients with PFT abnormalities compared to the group without (22% vs. 8%, respectively; *P *= 0.012). Significant coronary calcification (coronary artery calcium > 100) was more prevalent in patients with PFT abnormalities compared to those without abnormal PFTs (42% vs. 30%, respectively), but was not statistically significant (*P *= 0.14).

**Table 1:** Patient characteristics according to the presence of any pulmonary function abnormalities

Characteristic	No PFT abnormalities (n = 114)	PFT abnormalities (n = 45)	*P *value
Age (years)	61 ± 7.7	63 ± 9.9	0.14
Male gender	41 (36)	21 (47)	0.21
Caucasian	99 (87)	37 (82)	0.46
Body mass index (kg/m^2^)	29 ± 5.0	28 ± 5.5	0.12
Ever smoker	63 (55)	29 (64)	0.29
Current smoker	9 (7.9)	10 (22)	0.012
Reported lung disease	13 (11)	17 (38)	< 0.001
Emphysema	2 (1.8)	8 (18)	< 0.001
Asthma	12 (11)	8 (18)	0.22
Rheumatoid lung disease	0 (0)	2 (4.7)	0.07
CAC > 100	34 (30)	19 (42)	0.14
RA characteristics			
RA duration (years)	9.9 (5.6 to 18)	11 (7.1 to 23)	0.16
RF seropositive	64 (56)	35 (78)	0.011
Anti-CCP seropositive	67 (59)	37 (84)	0.003
DAS28 (units)	3.1 (2.4 to 3.7)	3.1 (2.4 to 4.2)	0.74
HAQ (units)	0.75 (0.13 to 1.5)	0.88 (0.13 to 1.5)	0.48
Modified Sharp Score (units)	41 (12 to 99)	56 (25 to 140)	0.098
Current prednisone	35 (31)	23 (51)	0.018
Current nonbiologic DMARDs	99 (88)	38 (84)	0.60
Current biologic DMARDs	55 (49)	20 (44)	0.63
Reported pulmonary symptoms			
Any reported symptoms	41 (36)	26 (58)	0.012
Any cough	19 (17)	16 (36)	0.010
Any phlegm	22 (19)	16 (36)	0.030
Any wheezing	17 (15)	18 (42)	0.001
Any breathlessness	18 (16)	15 (33)	0.014

Data expressed as mean ± standard deviation, n (%) or median (interquartile range). CAC, coronary artery calcium; CCP, cyclic citrullinated peptide; DAS28, RA Disease Activity Score in 28 joints; DMARDs, disease-modifying antirheumatic drugs; PFT, pulmonary function test; RA, rheumatoid arthritis; RF, rheumatoid factor.

Among RA characteristics, seropositivity for RF (*P *= 0.011) and for anti-CCP (*P *= 0.003), and current use of glucocorticoids (*P *= 0.018) were significantly higher in patients with PFT abnormalities. The specific diagnosis for which glucocorticoids were prescribed could not be determined. There were no significant differences in terms of RA disease activity or HAQ scores, but there is a trend toward a higher Sharp score (*P *< 0.06) in patients on corticosteroids. We find no difference in pulmonary symptoms between patients based on prednisone use. There is a significantly higher rate of prior diagnosis of emphysema in patients not on steroids (*P *< 0.001).

Patients with restriction had a statistically higher median C-reactive protein (*P *= 0.04) than the normal PFT group.

Pulmonary symptoms were reported in 78 patients (42%). Patients with PFT abnormalities were significantly more likely to report pulmonary symptoms (58% vs. 36%, respectively; *P *= 0.012) in all categories assessed (cough, phlegm, wheezing, and breathlessness).

### Association of pulmonary symptoms with patterns of pulmonary function test abnormalities

ROC curves for the association of individual pulmonary symptoms and PFT abnormalities are summarized in Table 2. Sensitivities and specificities are also summarized in Additional file 2. AUCs are depicted only for those models demonstrating internal validity. Within each category of symptoms, multiple symptoms were associated with any PFT abnormality but no single symptom resulted in AUC > 0.63, indicating that individual pulmonary symptoms alone were generally poor predictors of PFT abnormalities. Within each category of symptoms, frequent cough, chronic phlegm, frequent wheezing, and breathlessness after 100 yards of level walking demonstrated the highest AUCs for predicting abnormal PFTs and were used in subsequent multivariable modeling.

**Table 2:** Receiver operator characteristics for individual reported pulmonary symptoms to predict the presence of PFT abnormalities

Pulmonary symptom	Any PFT abnormality^a^		Restriction^a^		Obstruction^a^		Impaired diffusion^a^	
				
	AUC	95% CI^b^	AUC	95% CI^b^	AUC	95% CI^b^	AUC	95% CI^b^
Cough								
Frequent^c^	0.60	(0.53 to 0.67)^d^	-		-		0.61	(0.53 to 0.70)
Morning	0.57	(0.50 to 0.64)	-		0.62	(0.51 to 0.74)	0.65	(0.56 to 0.74)^d^
Daytime or night	0.57	(0.50 to 0.66)	-		-		0.59	(0.51 to 0.68)
Chronic^e^	-		-		0.62	(0.51 to 0.75)^d^	0.61	(0.52 to 0.71)
Phlegm								
Frequent^c^	-		-		-		0.59	(0.52 to 0.67)
Morning	-		-		-		0.60	(0.52 to 0.70)
Daytime or night	0.57	(0.51 to 0.63)	-		-		0.61	(0.53 to 0.70)
Chronic^e^	0.59	(0.53 to 0.67)^d^	-		-		0.66	(0.58 to 0.75)^d^
Wheezing								
Any	0.58	(0.53 to 0.65)	-		0.63	(0.51 to 0.76)^d^	0.62	(0.53 to 0.73)^d^
Often^f^	0.63	(0.56 to 0.71)^d^	-		-		-	
Breathlessness								
Hurrying or walking on incline	0.56	(0.51 to 0.63)	0.66	(0.51 to 0.80)	-		0.60	(0.51 to 0.70)^d^
With level walking at own pace	0.57	(0.51 to 0.64)	0.66	(0.51 to 0.81)^d^	-		0.59	(0.50 to 0.67)
After 100 yards of level walking	0.60	(0.52 to 0.67)^d^			-		-	

PFT, pulmonary function test. ^a^Areas under the curve (AUCs) are reported only for internally valid models; that is, those in which the 95% confidence interval (CI) did not include 0.5 (uninformative). ^b^95% CI obtained with repeated sampling and calculation of the AUC for 1, 000 bootstrap resamples with replacement. ^c^Defined as much as four to six times per day, 4 days or more per week. ^d^Symptoms selected for inclusion in subsequent multivariable modeling. ^e^Defined as on most days for at least 3 consecutive months per year. ^f^Defined as on most days or nights.

When specific PFT abnormalities were considered separately, breathlessness with level walking demonstrated the highest AUC for restriction (0.661, 95% CI = 0.509 to 0.828). For obstruction, both cough and wheezing were informative, with an AUC for chronic cough of 0.617 (95% CI = 0.512 to 0.746) and an AUC for any wheezing of 0.626 (95% CI = 0.510 to 0.761). For isolated impaired diffusion, symptoms of cough, phlegm, wheezing, and breathlessness all provided valid prediction, with AUCs for morning cough (0.646, 95% CI = 0.559 to 0.739), for chronic phlegm (0.658, 95% CI = 0.576 to 0.749), for any wheezing (0.623, 95% CI = 0.534 to 0.727), and for breathlessness with hurrying or walking on an incline (0.602, 95% CI = 0.513 to 0.699) demonstrating the highest values.

### Performance of combining pulmonary symptoms with patient characteristics to predict presence of any pulmonary function test abnormalities

Multivariable predictors of the presence of any PFT abnormalities were modeled, including the pulmonary symptoms identified from Table 2, along with other potential nonpulmonary predictors (Table 3). Among these, the predictors retained in the final model (Table 3, Model 3) included two pulmonary symptoms (chronic phlegm and breathlessness with walking 100 yards), patient report of a prior diagnosis of lung disease and four other characteristics (BMI, current smoking, seropositivity for anti-CCP antibodies, and current prednisone use), resulting in the following prediction equation:

**Table 3:** Multivariate associations of pulmonary symptoms, cardiac findings, and patient characteristics with any PFT abnormality

Characteristic	Model 1 (null)		Model 2 (complex)		Model 3^a ^(simplified)		*P *value
				
	OR	95% CI	OR	95% CI	OR	95% CI	
Frequent cough	1.61	(0.49 to 5.25)	2.05	(0.41 to 10.3)			
Chronic phlegm	2.64	(0.87 to 8.02)	3.01	(0.56 to 16.3)	4.16	(1.21 to 14.4)	0.02
Frequent wheezing	2.84	(0.74 to 10.9)	2.12	(0.37 to 12.1)			
Breathlessness at 100 yards	2.24	(0.60 to 8.32)	11.2	(1.54 to 81.6)	5.96	(1.25 to 28.3)	0.03
Reported diagnosis of pulmonary disease			4.57	(1.35 to 15.4)	3.77	(1.42 to 10.0)	0.01
CAC >100			1.25	(0.38 to 4.11)			
Age, per year			1.04	(0.97 to 1.12)			
Male vs. female			0.87	(0.27 to 2.84)			
White vs. other race			0.69	(0.20 to 2.31)			
Reported exercise, per quartile			0.95	(0.57 to 1.57)			
BMI, per kg/m^2^			0.91	(0.82 to 1.00)	0.93	(0.85 to 1.01)	0.08
Current smoking			5.34	(1.12 to 25.4)	4.01	(1.22 to 13.3)	0.02
Ever smoking vs. never smoking			0.73	(0.23 to 2.31)			
RA duration, per year			1.01	(0.95 to 1.07)			
RF seropositivity			1.53	(0.44 to 5.29)			
Anti-CCP seropositivity			2.99	(0.69 to 12.9)	2.82	(0.96 to 8.29)	0.06
DAS28, per unit			0.78	(0.45 to 1.33)			
HAQ, per unit			0.86	(0.35 to 2.10)			
Log-modified Sharp score, per log unit			1.16	(0.71 to 1.91)			
Current prednisone use			2.54	(0.95 to 6.85)	2.65	(1.12 to 6.25)	0.03
Current nonbiologic use			0.38	(0.08 to 1.88)			
Current biologic use			0.82	(0.27 to 2.52)			

Model 1 (null), pulmonary symptoms only; Model 2 (complex), multivariable model with extended covariate list; Model 3, multivariate model with covariates reduced based on hierarchical modeling. BMI, body mass index; CAC, coronary artery calcium; CCP, cyclic citrullinated peptide; CI, confidence interval; DAS28, RA Disease Activity Score in 28 joints; HAQ, Health Assessment Questionnaire; OR, odds ratio; PFT, pulmonary function test; RA, rheumatoid arthritis; RF, rheumatoid factor. ^a^*P *value for the likelihood ratio test comparing Models 2 and 3 = 0.801, indicating that the simpler model was not statistically different in predicting the variance in the outcome than the more complex model.

In this equation, for all predictors except BMI, a value of 1 would be entered for a patient with the predictor; otherwise, 0 would be entered. For BMI, the actual value of the patient's BMI would be used.

**Figure 1:**
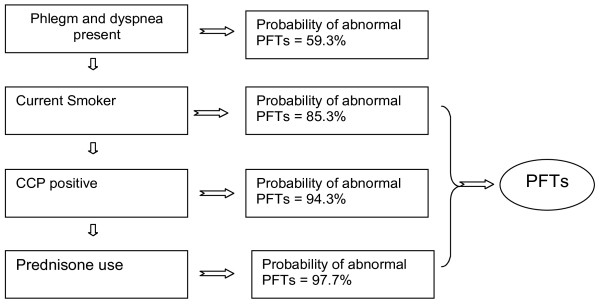
**Schematic of screening for a patient with rheumatoid arthritis and without known lung disease**. Application of the described algorithm to a model patient demonstrates how the probability of pulmonary function test (PFT) abnormality varies with specific patient features. See Additional file 3 for calculated probabilities based on the presence of different combinations of predictors. CCP, cyclic citrullinated peptide.

The AUC for the ROC curve including only the two significant pulmonary symptoms was 0.612 (95% CI = 0.529 to 0.693; Table 4), indicating that the combination of symptoms was only slightly better than each individually in predicting any abnormal PFTs. Addition of current smoking, BMI, anti-CCP seropositivity, and current prednisone use to the model increased the AUC to 0.773 (95% CI = 0.679 to 0.857). This ROC curve obtained from the extended model was statistically different from the symptom-only model (*P *= 0.0006). Although adding patient report of lung disease to the extended model increased the AUC of the ROC curve slightly (0.799, 95% CI = 0.709 to 0.873), the ROC curves did not statistically differ (*P *= 0.26). Similarly, exclusion of patients with a prior diagnosis of lung disease did not alter the performance of the extended model compared with the full cohort. Based on the algorithm developed, a schematic for identifying patients for screening with PFTs is provided in Figure 1 and the probabilities for abnormal PFTs given different combinations of predictors are outlined in Additional file 3.

**Table 4:** Receiver operator curve validation of multivariable models predicting the presence of any abnormal PFTs

	Full cohort (n = 159)			Cohort excluding reported lung disease (n = 129)		
		
	AUC	95% CI^a^	*P *value	AUC	95% CI^a^	*P *value
Symptoms only^b^	0.61	(0.53 to 0.69)	-	0.64	(0.55 to 0.76)	-
Symptoms + other factors^c^	0.77	(0.68 to 0.86)	0.0006*	0.79	(0.67 to 0.89)	0.005*
Symptoms + factors + lung disease^d^	0.80	(0.71 to 0.87)	0.26**	-	-	-

PFT, pulmonary function test. ^a^95% confidence interval (CI) obtained with repeated sampling and calculation of area under the curve (AUC) for 1,000 bootstrap resamples with replacement. ^b^Symptoms included were those from the final predictive multivariable model (chronic phlegm and breathlessness with ambulating 100 yards). ^c^Other factors included were those from the final predictive multivariable model (current smoking, body mass index, presence of anti-cyclic citrullinated peptide antibodies, and current prednisone use). ^d^Lung disease includes patient self-report of emphysema, asthma, and rheumatoid lung disease. *P values represent the comparison of AUC functions for the symptoms + other factors model vs. the symptom-only model. **P values represent the comparison of AUC functions for the symptoms + factors + lung disease model vs. the symptoms + factors model.

We performed multivariable models for the prediction of specific PFT abnormalities (restriction, obstruction, impaired diffusion) (Additional file 4). For restriction, one symptom (breathlessness with level walking) and two patient characteristics (BMI and current prednisone use) were retained in the final prediction equation. The AUC for the ROC curve for the model including all three predictors was 0.786 (95% CI = 0.585 to 0.918) (Table 5) and differed significantly from the ROC curve that included pulmonary symptoms only (*P *= 0.046).

**Table 5:** Receiver operator curve validation of multivariable models predicting the presence of specific PFT abnormalities

Characteristics included in model	Full cohort (n = 159)			Cohort excluding reported lung disease (n = 129)		
		
	AUC	95% CI^a^	*P *value	AUC	95% CI^a^	*P *value
Model predicting restriction						
Pulmonary symptoms only	0.66	(0.51 to 0.81)	-	0.63	(0.51 to 0.80)	-
Symptoms + other factors^b^	0.79	(0.59 to 0.92)	0.046*	0.79	(0.61 to 0.92)	0.040*
Model predicting obstruction						
Pulmonary symptoms only	0.62	(0.50 to 0.74)	-	0.62	(0.51 to 0.75)	-
Symptoms + other factors^c^	0.91	(0.78 to 0.98)	0.0001*	0.86	(0.75 to 0.94)	0.0005*
Symptoms + factors + lung disease	0.96	(0.91 to 0.99)	0.080**	-	-	-
Model predicting impaired diffusion						
Pulmonary symptoms only	0.66	(0.58 to 0.73)	-	0.66	(0.57 to 0.75)	-
Symptoms + other factors^d^	0.85	(0.75 to 0.93)	0.0003*	0.85	(0.73 to 0.93)	0.001
Symptoms + factors + lung disease	0.86	(0.74 to 0.94)	0.41**	-	-	-

PFT, pulmonary function test. ^a^95% confidence interval (CI) obtained with repeated sampling and calculation of area under the curve (AUC) for 1,000 bootstrap resamples with replacement. ^b^Other factors included were those from the final predictive multivariable model (body mass index and current prednisone use). ^c^Other factors included were those from the final predictive multivariable model (gender, exercise, current smoking, body mass index, presence of rheumatoid factor, and current prednisone use). ^d^Other factors included were those from the final predictive multivariable model (age, current smoking, body mass index, and current prednisone use). *P values represent the comparison of AUC functions for the symptoms + other factors model vs. the symptom-only model. **P values represent the comparison of AUC functions for the symptoms + factors + lung disease model vs. the symptoms + factors model.

For obstruction, eight predictors were retained in the final model (Additional file 4), including one symptom (chronic cough), prior diagnosis of lung disease, and six other characteristics (gender, exercise, BMI, current smoking, RF seropositivity, and current prednisone use). The AUC for the ROC curve including chronic cough, gender, exercise, BMI, smoking, and prednisone use was 0.905 (95% CI = 0.784 to 0.978) (Table 5) and was significantly higher than the model including only chronic cough (*P *= 0.0001). Addition of reported prior lung disease to the extended prediction model increased the AUC to 0.961 (95% CI = 0.908 to 0.992); however, the difference between the ROC curves was not statistically significant (*P *= 0.080).

For impaired diffusion, six predictors were retained in the final model (Additional file 4), including one symptom (chronic phlegm), prior diagnosis of lung disease, and four other characteristics (age, BMI, current smoking, and current prednisone use). The AUC for the extended model including chronic phlegm, age, BMI, smoking, and current prednisone was 0.852 (95% CI = 0.749 to 0.934) (Table 5), and differed significantly from the model including chronic phlegm only (*P *= 0.0003). The addition of reported pulmonary disease was associated with a slight increase in AUC (0.864, 95% CI = 0.737 to 0.936), but was not significantly different than the model not including reported pulmonary disease.

For all models, AUCs for the ROC curves were not substantially reduced when the cohort was restricted to patients who did not report a prior diagnosis of pulmonary disease (Table 5).

## Discussion

Timely recognition of the pulmonary manifestations of RA is critical in light of the fact that respiratory involvement is identified as the second leading cause of mortality in patients with RA. Pulmonary disease in RA patients may not be routinely sought by rheumatologists and internists in the absence of cost-effective, accurate and time-efficient means of screening. An adequate screening tool that could easily be integrated into clinical care would represent a critical step in early identification and treatment of these conditions. In the present study, we demonstrated that pulmonary symptoms in combination with other easily measured variables can predict PFT abnormalities in patients with RA, and can identify patients in greatest need of further workup.

Nearly one-third of subjects in this prospective RA cohort demonstrated PFT abnormalities, and the majority of these patients carried no prior pulmonary diagnosis. As might be expected, respiratory symptoms were statistically more common in patients with abnormal PFTs. Specific symptoms in combination with commonly assessed patient characteristics were highly predictive of PFT abnormalities. We conclude that, beyond smoking, factors such as positive RF and anti-CCP antibodies and ongoing corticosteroid treatment may, in combination with pulmonary symptoms, identify individuals in need of further pulmonary evaluation. A simplified schematic of this screening approach (Figure 1) indicates how these findings might guide subsequent evaluation with PFTs.

The findings of our study are supported by prior studies that have identified individual factors in association with lung disease. High titers of anti-CCP antibodies have been associated with the presence of pulmonary fibrosis in patients with RA [42]. The association of corticosteroid use with PFT abnormalities could be related to the effects of the drug itself or, more probably, identifies patients with severe or difficult to treat RA, a known risk factor for PFT abnormalities [16,43]. In the present study we were unable to determine the diagnosis for which corticosteroids were prescribed. Corticosteroid use may therefore be a marker of RA disease activity or a marker of lung disease.

Previous studies have reported association of symptoms and/or patient and disease characteristics with lung disease in RA patients. Our results are not directly comparable given differences in primary outcome and targeted population. Dawson and colleagues [10] detected no difference in the prevalence of respiratory symptoms (dyspnea New York Heart Association grades II and III and productive cough) in 28 patients with HRCT-documented ILD compared to 122 patients without [10]. Gabbay and colleagues *a priori *divided 36 early RA patients based on symptoms, PFT and HRCT results, but did not test predictor variables against a uniform outcome [12]. The study by Gochuico and colleagues enrolled 64 RA patients without respiratory symptoms [17], and thus was primarily focused on identifying prevalence and predictors of pulmonary disease in asymptomatic RA patients. A number of isolated factors have been found to associate with ILD in RA patients, including cigarette smoking, male gender, higher HAQ score, genetic predisposition, the presence of other extra-articular manifestations of RA and treatment with methotrexate [6,12,17,44-46].

In contrast to previous studies, ours is the first study to exclude patients with clinically apparent CVD, thus increasing the likelihood that the reported respiratory symptoms truly reflected lung disease, rather than cardiac disease. Furthermore, to control for confounding by subclinical CVD, we adjusted for the severity of subclinical CAD in our analyses. We found no statistically significant association between subclinical CAD and PFT abnormalities in our population. Furthermore, the association between pulmonary symptoms and PFT abnormalities was not affected after adjusting for subclinical CAD.

The present study differs from previously described cohorts in a number of other important matters. Because this analysis is nested in a larger ongoing natural history study, our patient cohort is extensively characterized with available demographic, lifestyle and anthropometric information. The number of subjects is considerably larger than in previous studies. Measures of functionality (beyond the HAQ score) are available, including accurate recording of exercise. Patients with RA may be physically deconditioned, and this may interfere with recognition of respiratory symptoms. The information available in our study allowed us to address this potential confounder as both HAQ scores and reported exercise were included and neither demonstrated a significant effect in multivariable modeling.

There are some notable limitations in our study. PFTs are not the gold standard for detecting respiratory disease. We chose to use PFTs rather than computed tomography scans as our marker of lung disease in this analysis as they provide a common and low-risk diagnostic modality that often precedes radiographic evaluation in clinical practice. We believe that using a more sensitive imaging method might strengthen our associations by identifying parenchymal abnormalities in patients who reported symptoms but were found to have normal PFTs. The small number of patients with lung disease in this cohort results in large confidence intervals in multivariate analysis. This points to the need for replication of these findings in a larger patient population.

There may have been an unintentional selection bias against patients with significant lung disease who may have elected not to participate in our study. In this case, however, we would expect to observe stronger associations between symptoms and PFT abnormalities had such patients participated. Furthermore, in the current study, coronary artery scanning occurred 1.3 to 2.5 years before pulmonary function testing. This might have lead to an underestimation of coronary disease that developed in the intervening time. Finally, our results may not be completely applicable in practice where RA patients with clinically significant CVD are more common. In a population with a higher frequency of CVD, respiratory symptoms may be less specific to pulmonary disease. This can only be addressed by a larger study in a population not restricted for clinically significant CVD.

## Conclusions

In summary, we observed a high prevalence of PFT abnormalities in a selected population of RA patients and a considerable frequency of respiratory symptoms as assessed by a formal questionnaire. Respiratory symptoms and specific patient characteristics were identified as predictors of lung disease as determined by PFTs. It has been suggested that early identification and timely therapeutic intervention with antifibrotic agents may alter the prognosis of pulmonary fibrosis [17,46,47]. Similarly, early intervention in patients with RA and chronic obstructive pulmonary disease might improve quality of life and performance status. A practical, cost-effective way of identifying early pulmonary disease in patients with RA could yield significant benefit in patient outcomes. This study suggests a limited set of questions could be incorporated into clinical practice that would provide guidance regarding which patients should undergo subsequent pulmonary function testing and radiographic imaging. These findings may be of clinical benefit if confirmed in a larger population study.

## Abbreviations

AUC: area under the curve; BMI: body mass index; CAD: coronary artery disease; CCP: cyclic citrullinated peptide; CI: confidence interval; CVD: cardiovascular disease; ELISA: enzyme-linked immunosorbent assay; HAQ: Health Assessment Questionnaire; HRCT: high-resolution computed tomography; ILD: interstitial lung disease; PFT: pulmonary function test; RA: rheumatoid arthritis; RF: rheumatoid factor; ROC: receiver operator curve; TNF: tumor necrosis factor.

## Competing interests

The authors declare that they have no competing interests.

## Authors' contributions

DAP participated in the study design, data collection and analysis as well as in writing and editing the manuscript. JTG performed statistical analyses and edited the manuscript. GC participated in data collection and edited the manuscript. NL performed statistical analysis and edited the manuscript. JMB participated in the study design as well as drafting and editing the manuscript. SKD participated in the study design, and drafted and edited the manuscript.

## Supplementary Material

Additional file 1 **Lung Tissue Research Consortium Questionnaire**. Word document containing the questionnaire developed by the Lung Tissue Research Consortium for assessment of pulmonary symptoms. Click here for file

Additional file 2 **Sensitivity, specificity, positive predictive value and hegative predictive value of individual reported pulmonary symptoms for the presence of PFT abnormalities**. Word document containing sensitivity, specificity, positive predictive value and negative predictive value of individual reported pulmonary symptoms for the presence of any PFT abnormality, restriction, obstruction or impaired diffusion. Click here for file

Additional file 3 **Probability of abnormal PFTs with varying combinations of predictors**. Word document containing a graphical representation of the range of probabilities associated with various combinations of patient variables. Click here for file

Additional file 4 **Multivariable models for prediction of specific PFT abnormalities**. Word document containing multivariable models for prediction of specific PFT abnormalities including restriction, obstruction or decreased diffusion capacity of the lung for carbon monoxide. Click here for file
