# Supplementary figures and images for: Respiratory symptoms and disease characteristics as predictors of pulmonary function abnormalities in patients with rheumatoid arthritis: an observational cohort study
Source: Arthritis Res Ther. 2010 May 27;12(3):R104. doi: 10.1186/ar3037 (PMC2911894; doi:10.1186/ar3037)

**Additional Data File 1:**


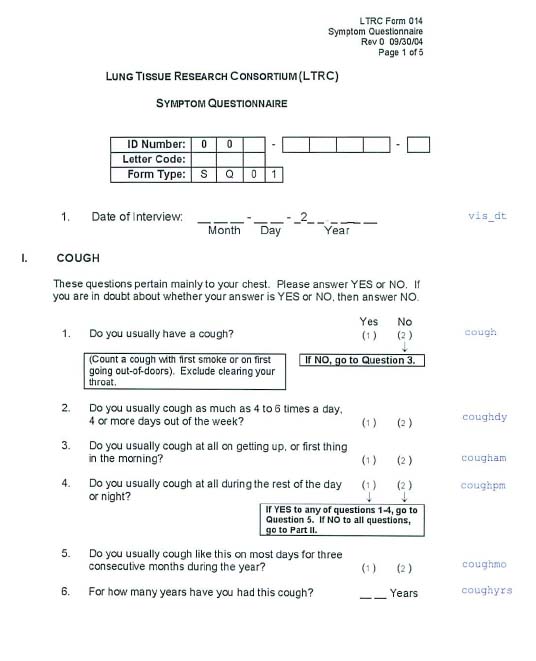


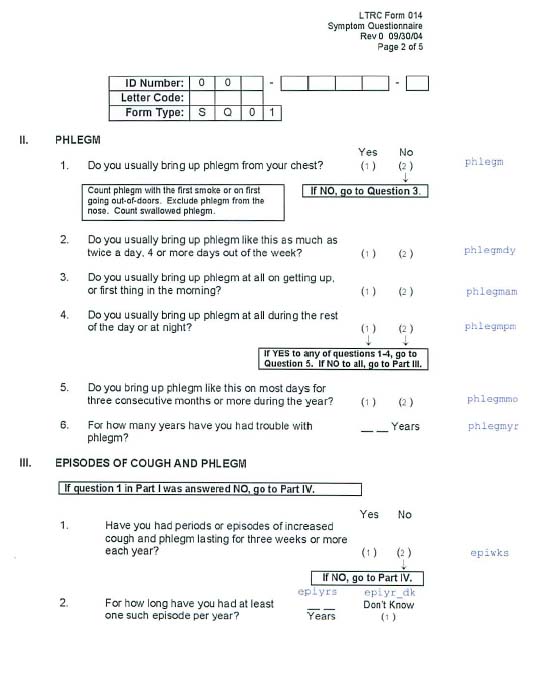


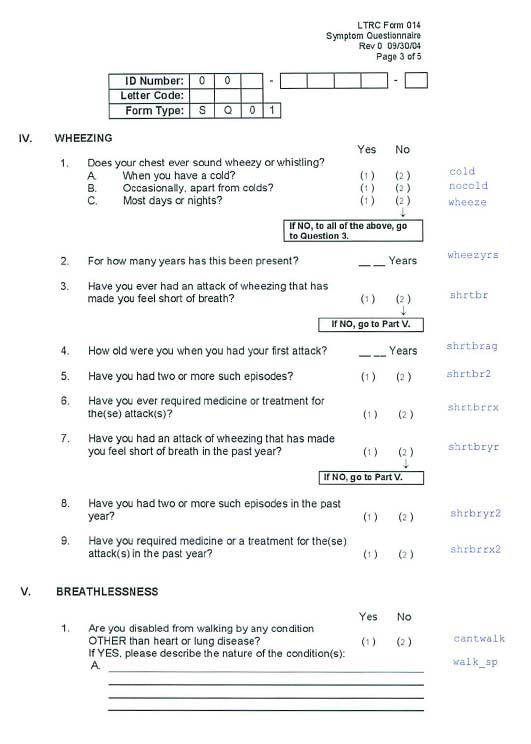


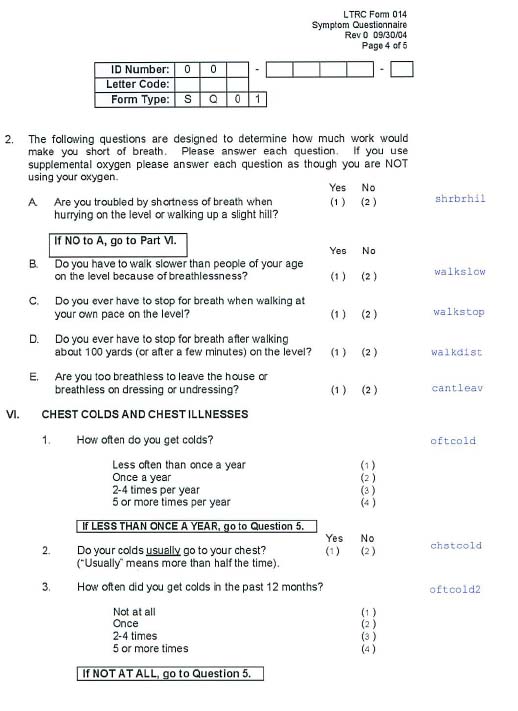


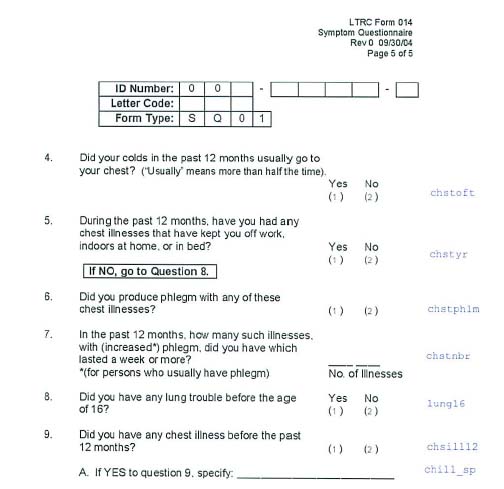

Supplement: Additional file 1 — Lung Tissue Research Consortium Questionnaire. Word document containing the questionnaire developed by the Lung Tissue Research Consortium for assessment of pulmonary symptoms. [file ar3037-S1.DOC]
