# Supplementary material for: Respiratory symptoms and disease characteristics as predictors of pulmonary function abnormalities in patients with rheumatoid arthritis: an observational cohort study
Source: Arthritis Res Ther. 2010 May 27;12(3):R104. doi: 10.1186/ar3037 (PMC2911894; doi:10.1186/ar3037)
Supplement: Additional file 2 — Sensitivity, specificity, positive predictive value and hegative predictive value of individual reported pulmonary symptoms for the presence of PFT abnormalities. Word document containing sensitivity, specificity, positive predictive value and negative predictive value of individual reported pulmonary symptoms for the presence of any PFT abnormality, restriction, obstruction or impaired diffusion. [file ar3037-S2.DOC]

Appendix 2. Sensitivity, Specificity, PPV and NPV of Individual Reported Pulmonary Symptoms for the Presence of PFT Abnormalities

________________________________________________________________________________________________________________________________________

Pulmonary Symptom Any PFT Abnormality Restriction Obstruction Impaired Diffusion

Sn, Sp, PPV, NPV Sn, Sp, PPV, NPV Sn, Sp, PPV, NPV Sn, Sp, PPV, NPV ________________________________________________________________________________________________________________________________________

*Cough*

Frequent * 22.7, 91.2, 50.0, 75.4 25.0, 88.4, 15.0, 93.5 29.4, 89.4, 25.0, 91.3 30.0, 92.1, 47.4, 84.7

Morning 27.3, 93.0, 60.0, 76.8 25.0, 88.4, 15.0, 93.5 35.3, 90.1, 30.0, 92.0 36.7, 93.7, 57.9, 86.1

Daytime or Night 29.5, 86.0, 44.8, 76.0 33.3, 82.9, 13.8, 93.8 35.3, 83.7, 20.7, 91.5 33.3, 85.7, 35.7, 84.4

Chronic^**^ 29.5, 86.0, 44.8, 76.0 25.0, 82.2, 10.3, 93.0 41.2, 84.4, 24.1, 92.2 36.7, 86.5, 39.3, 85.2

*Phlegm*

Frequent * 15.9, 94.7, 53.8, 74.5 16.7, 92.5, 15.4, 93.1 11.8, 92.2, 15.4, 89.7 23.3, 95.2, 53.8, 83.9

Morning 25.0, 86.8, 42.3, 75.0 16.7, 83.6, 7.69, 92.4 23.5, 84.4, 15.4, 90.2 33.3, 87.3, 38.5, 84.6

Daytime or Night 20.5, 93.0, 52.9, 75.2 25.0, 90.4, 17.6, 93.6 17.6, 90.1, 17.6, 90.1 30.0, 93.7, 52.9, 84.9

Chronic^**^ 27.3, 92.1, 57.1, 76.6 25.0, 87.7, 14.3, 93.4 17.6, 87.2, 14.3, 89.8 40.0, 92.9, 57.1, 86.7

*Wheezing*

Any 39.5, 85.8, 51.5, 78.9 33.3, 79.9, 12.1, 93.5 43.8, 81.4, 21.2, 92.7 41.4, 83.2, 36.4, 86.0

Often^***^ 20.9, 95.6, 64.3, 76.1 25.0, 92.4, 21.4, 93.7 18.8, 92.1, 21.4, 90.8 20.7, 93.6, 42.9, 83.6

*Breathlessness*

Hurrying or walking on incline 34.9, 84.2, 45.5, 77.4 50.0, 81.4, 18.2, 95.2 25.0, 79.4, 12.1, 90.3 37.9, 82.5, 33.3, 85.2

With level walking at own pace 22.7, 92.1, 52.6, 75.5 41.7, 90.4, 26.3, 95.0 11.8, 87.9, 10.5, 89.2 26.7, 91.3, 42.1, 83.9

After 100 yards of level walking 18.2, 94.7, 57.1, 75.0 25.0, 92.5, 21.4, 93.8 11.8, 91.5, 14.3, 89.6 20.0, 93.7, 42.9, 83.1

* Defined as much as 4 to 6 times per day, 4 or more days per week

** Defined as on most days for at least 3 consecutive months per year

*** Defined as on most days or nights
