# Supplementary material for: Respiratory symptoms and disease characteristics as predictors of pulmonary function abnormalities in patients with rheumatoid arthritis: an observational cohort study
Source: Arthritis Res Ther. 2010 May 27;12(3):R104. doi: 10.1186/ar3037 (PMC2911894; doi:10.1186/ar3037)
Supplement: Additional file 3 — Probability of abnormal PFTs with varying combinations of predictors. Word document containing a graphical representation of the range of probabilities associated with various combinations of patient variables. [file ar3037-S3.DOC]

**Additional Data File 3**

**APPENDIX 3**. Predicted probabilities of any PFT abnormality based on sample combinations of predictors

Predictor Combination 1 Combination 2 Combination 3 Combination 4 Combination 5 Combination 6 Combination 7 Combination 8
__________________________________________________________________________________________________________________________________________________________________

Chronic phlegm + + + + + + + +

Breathlessness - + + + + + + +

Current smoking - - + + + + - -

BMI of 25 + + + + + + + +

Anti- CCP positive - - - + + + + +

Current prednisone - - - - + + + -

Reported lung disease - - - - - + - -

Predicted probability 19.4% 59.3% 85.3% 94.3% 97.7% 99% 91.5% 80.4%
