# Supplementary material for: Respiratory symptoms and disease characteristics as predictors of pulmonary function abnormalities in patients with rheumatoid arthritis: an observational cohort study
Source: Arthritis Res Ther. 2010 May 27;12(3):R104. doi: 10.1186/ar3037 (PMC2911894; doi:10.1186/ar3037)
Supplement: Additional file 4 — Multivariable models for prediction of specific PFT abnormalities. Word document containing multivariable models for prediction of specific PFT abnormalities including restriction, obstruction or decreased diffusion capacity of the lung for carbon monoxide. [file ar3037-S4.DOC]

**Additional Data File 4:**

**APPENDIX 4.** Multivariate Associations of Pulmonary Symptoms and Patient Characteristics with Specific PFT Abnormalities

______________________________________________________________________________________________________________________________________

Characteristic Restriction Obstruction Diffusion Defect

OR 95% CI p OR 95% CI p OR 95% CI p

______________________________________________________________________________________________________________________________________

Chronic Cough 11.85 (1.47 – 95.68) 0.020

Chronic Phlegm 23.32 (5.18 – 105.0) <0.001

Breathlessness with level walking 6.16 (1.33 – 20.02) 0.018

Reported pulmonary disease 41.64 (4.99 – 347.2) 0.001 3.68 (1.02 – 13.28) 0.047

Age, per year 1.11 (1.04 – 1.19) 0.003

Male vs. female 0.09 (0.01 – 1.06) 0.056

Reported exercise, per quartile 5.57 (1.64 – 18.90) 0.006

BMI, per kg/m2 1.11 (1.00 – 1.24) 0.059 0.75 (0.60 – 0.93) 0.009 0.88 (0.79 – 0.99) 0.030

Current smoking 22.39 (1.76 – 285.1) 0.017 20.81 (4.80 – 90.11) <0.001

RF seropositivity 53.48 (2.60 – 1096.4) 0.010

Current prednisone use 4.31 (1.12 – 16.62) 0.034 16.11 (1.95 – 133.15) 0.010 2.95 (0.96 – 9.07) 0.060
